# The temporal dynamics of the tracheal microbiome in tracheostomised patients with and without lower respiratory infections

**DOI:** 10.1371/journal.pone.0182520

**Published:** 2017-08-10

**Authors:** Marcos Pérez-Losada, Robert J. Graham, Madeline Coquillette, Amenah Jafarey, Eduardo Castro-Nallar, Manuel Aira, Robert J. Freishtat, Jonathan M. Mansbach

**Affiliations:** 1 Computational Biology Institute, Milken Institute School of Public Health, George Washington University, Washington, DC, United States of America; 2 Division of Emergency Medicine, Children’s National Medical Center, Washington, DC, United States of America; 3 CIBIO-InBIO, Universidade do Porto, Campus Agrário de Vairão, Vairão, Portugal; 4 Division of Critical Care Medicine, Department of Anesthesiology, Perioperative, and Pain Medicine, Boston Children's Hospital, Harvard Medical School, Boston, Massachusetts, United States of America; 5 Department of Medicine, Boston Children's Hospital, Harvard Medical School, Boston, Massachusetts, United States of America; 6 Center for Bioinformatics and Integrative Biology, Facultad de Ciencias Biológicas, Universidad Andrés Bello, Santiago, Chile; 7 Departamento de Ecoloxía e Bioloxía Animal, Universidade de Vigo, Vigo, Spain; University of Illinois at Urbana-Champaign, UNITED STATES

## Abstract

**Background:**

Airway microbiota dynamics during lower respiratory infection (LRI) are still poorly understood due, in part, to insufficient longitudinal studies and lack of uncontaminated lower airways samples. Furthermore, the similarity between upper and lower airway microbiomes is still under debate. Here we compare the diversity and temporal dynamics of microbiotas directly sampled from the trachea via tracheostomy in patients with (YLRI) and without (NLRI) lower respiratory infections.

**Methods:**

We prospectively collected 127 tracheal aspirates across four consecutive meteorological seasons (quarters) from 40 patients, of whom 20 developed LRIs and 20 remained healthy. All aspirates were collected when patients had no LRI. We generated 16S rRNA-based microbial profiles (~250 bp) in a MiSeq platform and analyzed them using Mothur and the SILVAv123 database. Differences in microbial diversity and taxon normalized (via negative binomial distribution) abundances were assessed using linear mixed effects models and multivariate analysis of variance.

**Results and discussion:**

Alpha-diversity (ACE, Fisher and phylogenetic diversity) and beta-diversity (Bray-Curtis, Jaccard and Unifrac distances) indices varied significantly (P<0.05) between NLRI and YLRI microbiotas from tracheostomised patients. Additionally, *Haemophilus* was significantly (P = 0.009) more abundant in YLRI patients than in NLRI patients, while *Acinetobacter*, *Corynebacterium* and *Pseudomonas* (P<0.05) showed the inverse relationship. We did not detect significant differences in diversity and bacterial abundance among seasons. This result disagrees with previous evidence suggesting seasonal variation in airway microbiotas. Further study is needed to address the interaction between microbes and LRI during times of health and disease.

## Introduction

The population of children with a tracheostomy is increasing [1] and these children are at high risk of developing lower respiratory infections (LRI) [2] that require intensive care [3]. A retrospective analysis of 917 children aged 0–18 years from 36 children's hospitals who received a tracheostomy in 2002 demonstrated that in the 5-year follow-up period, the mean number of hospitalizations experienced per child was 3.8 (SD: 4.4; range: 0–34) and 46% of the hospitalizations were for “respiratory” diagnoses [2].

Despite this risk of LRI, there is no consensus about how providers should treat common acute respiratory infections in tracheostomised children [3, 4]. While common respiratory viruses and bacteria in the airway tract are identifiable utilizing standard laboratory techniques and have provided interesting avenues for research (e.g., [5]), these common organisms may only represent a small fraction of the commensal and pathogenic microbes living in the airways of an individual [6]. Indeed, the traditional conceptual model of these potentially life-threatening LRIs (i.e., pathogen causing disease in a sterile lung) may be too simplistic [7]. More current conceptual models of LRIs account for the highly functional bacterial communities (i.e., microbiota) that are present in the upper and lower airways (nose to lung) [8–13] and influence both immune [14–18] and inflammatory responses [18, 19].

However, the role that the airway microbiota plays during LRI is still poorly understood since: i) airway microbiome research focuses mainly on the nasopharynx and oral cavity and far less on the lower airways due to easier access and ethical considerations [20], and ii) most microbiome studies during both health and disease are cross-sectional [21–26] and neglect the temporal dynamics of the microbial communities [11]. Furthermore, upper airway samples are frequently used as a proxy for the lower airway, but the similarity of lower and upper airway microbiotas during disease is still under debate [27, 28]. Hence, since studying the dynamics of lower respiratory microbiotas during both health and disease (e.g., infection) is a challenging endeavor, new approaches or models [29] are needed to continue their investigation.

We have addressed these knowledge gaps by directly examining the dynamics of tracheal microbiotas (i.e., bypassing the nose and mouth) in a longitudinal study of 40 tracheostomised children and young adults with and without LRIs. We aimed to determine the differences in the microbiotas of these patients and their variation over one calendar year. We hypothesized that tracheal microbiotas differ in composition and structure according to whether or not individuals developed LRIs.

## Materials and methods

### Ethics

This study was approved by the Boston Children's Hospital Institutional Review Board (IRB), which requires that consent is obtained and documented prior to conducting study procedures and collection of samples for research. Written consent was obtained from all independent participants or their legal guardians using the Boston Children's Hospital IRB approved informed consent documents (IRB No P00007853).

### Cohort

The Critical Care, Anesthesia, Perioperative Extension (CAPE) and Home Ventilation Program began in June 2007 for children and young adults with respiratory technology dependence in an effort to enhance outpatient and transitional-care services [30–32]. The participants’ diagnoses include, but are not limited to, spinal muscular atrophy, spastic quadriplegia with respiratory insufficiency, muscular dystrophy, mitochondrial disorders, spinal cord injuries, idiopathic hypotonia, and a range of complex chronic lung disease. All participants in this study resided in New England (USA) at the time of sampling.

### Sample collection and storage

Samples were collected every meteorological season or quarter (Q) over the course of one calendar year starting in the fall of 2013 and ending in the summer of 2014: Q1 (fall) = 10/2013 to 11/2013, Q2 (winter) = 12/2013 to 02/2014, Q3 (spring) = 03/2014 to 05/2014 and Q4 (summer) = 06/2014 to 08/2014. All samples were collected when patients were healthy (i.e., no symptoms of LRI for previous four weeks), since our goal was to determine whether microbiotas from individuals who develop a LRI are different from those who do not develop a LRI. A LRI was defined as any illness causing increased mucus production and requiring increased oxygen delivery or higher ventilator settings over baseline. In order to ensure standardized sample collection, we observed parents or visiting nurses collecting the first sample in person during a home or clinic visit. The aspirate was stored at 0°C within 15 minutes of being collected. The samples were picked up by the study team, transported on ice to Boston Children’s Hospital, and stored at -80°C.

### High-throughput sequencing

We aimed to sequence one tracheal aspirate sample per quarter (four quarters total) from both participants who acquired a LRI (yes LRI = YLRI) during the study and those who remained healthy (no LRI = NLRI), hence rendering four samples per participant. Q1 to Q4 samples in both YLRI and NLRI patients are isochronous. Total DNA was extracted using the QIAGEN QIAamp DNA Kit (Catalog # 51304). All samples were incubated in 200 μL of lysozyme-TE buffer pH = 8.0 for 30 minutes at 37°C. All extractions yielded >5 ng/μL of total DNA (as indicated by NanoDrop 2000 UV-Vis Spectrophotometer measuring). DNA extractions were prepared for sequencing using the Schloss’ MiSeq_WetLab_SOP protocol [33]. Each DNA sample was amplified for the V4 region (~250 bp) of the 16S rRNA gene and all libraries were sequenced together in a single run of the Illumina MiSeq sequencing platform at University of Michigan Medical School. Negative controls processed as above showed no PCR band on an agarose gel.

### Sequence and statistical analyses

Raw FASTQ files were processed in mothur v1.35.1 [34] and indicated in the MiSeq SOP (www.mothur.org/wiki/MiSeq_SOP). Default settings were used to minimize sequencing errors [35]. Clean sequences were aligned to the SILVA123-based bacterial reference alignment at www.mothur.org. Chimeras were removed using uchime [36] and non-chimeric sequences were classified using a naïve Bayesian classifier [37]. Sequences were clustered into Operational Taxonomic Units (OTUs) at the 0.03 similarity threshold (species level). A consensus taxonomy was generated based on the classification of sequences clustered within an OTU. OTU sequence representatives and taxonomy were then converted to a BIOM file for subsequent analyses and all OTU singletons (n = 1) were eliminated. We normalized our samples using the negative binomial distribution as recommended by McMurdie and Holmes [38] and implemented in the Bioconductor package DESeq2 [39]. This approach simultaneously accounts for library size differences and biological variability. Microbial normalized counts generated this way are referred to as taxon abundances throughout the text. Trees for phylogenetic diversity calculations were constructed using FastTree and midpoint rooting [40].

Taxonomic alpha-diversity was estimated using Shannon, Fisher and ACE indices, while phylogenetic alpha-diversity was calculated by the Faith’s phylogenetic diversity index [41]. Beta-diversity was estimated using phylogenetic Unifrac (unweighted and weighted), Bray-Curtis and Jaccard distances. Dissimilarity between samples was explored using principal coordinates analysis (PCoA). Linear mixed-effects (LME) models analysis, as implemented in the lmer4 R package [42], was applied to both alpha-diversity indices and taxa (genera and phyla) abundances (response) while accounting for non-independence of subjects (random effect) and LRI (predictor). Time was modeled as “number of days since the collection of the first sample” (days). Additionally, we were also interested in looking at microbial variation across meteorological seasons (quarters) because microbial epidemics and patients usually change their behavior through seasons and because previous studies (e.g., [11, 43, 44]) showed significant associations between microbial variation and season. Hence, we also included meteorological seasons or quarters in our models as either a numerical or a categorical variable. We performed multiple rounds of analysis including all of these factors and the co-variables in Table 1 and S1 Table (age, gender, feeding route, ventilator use, oxygen requirement, tracheostomy change frequency, prophylactic antibiotics and daily inhaled steroids). We also tested the interaction between LRI and Time and between LRI and Quarters. We also compared models with random intercepts and random slopes and the order of our factors. Initial LME models were compared using the function lmerTest, which performs automatic backward elimination of factors. ANOVA type II and III (if interactions were included in the model) tests were also carried out for hypothesis testing. Model assumptions in final LME models were validated using residual vs fit plots and a normal probability plots. Beta-diversity Unifrac indices were compared using permutational multivariate analysis of variance (adonis) as implemented in the vegan R package [45]. Adonis models were compared using the Akaike Index Criterion [46]. We treated our random factors as fixed factors and put them first in the model. Significance was determined through 10,000 permutations. Our preliminary analyses showed that random slopes, Time (alone or interacting with LRI), Quarters (alone or interacting with LRI) and all co-variables in Table 1, except daily inhaled steroids, did not have a significant impact on any representation of microbial diversity or taxon abundance. Hence, our final (most parsimonious) LME and adonis models and analyses included one predictor (LRI) and one co-variable (daily inhaled steroids). Bonferroni or Benjamini-Hochberg FDR multiple test correction methods were applied. All analyses were performed in mothur, QIIME [47], R [48] and RStudio [49].

**Table 1:** Clinical characteristics of the studied cohort. NLRI = patients with no lower respiratory infections. YLRI = patients with lower respiratory infections.

Variables	NLRI (N = 20)	YLRI (N = 20)	P-value
Years of Age, median (range)	8.5 (<1–30)	12.5 (<1–34)	0.342
Daily Inhaled Steroids	6	12	0.068
Feeding Route			0.210
G-tube	12	7	
GJ-tube	4	2	
Oral	2	2	
Oral & G-tube	2	9	
Gender Male	15	14	0.999
Oxygen Requirement	3	8	0.090
Prophylactic Antibiotics	5	2	0.408
Multiple Tracheostomy Changes	9	6	0.514
Ventilator Use			0.113
Continuous	14	7	
Night/Nap	4	11	
No	2	2	

Clinical characteristics were compared between NLRI and YLRI patients using Fisher’s exact, or Mann-Whitney tests, as appropriate.

## Results

Forty patients were enrolled in this study of whom 20 had at least one clinically-evident LRI (YLRI) and 20 had no LRI (NLRI). Clinical characteristics for the study cohort are presented in Table 1 and S1 Table. When “healthy” or at their clinical baseline, we collected 62 tracheal samples from YLRI participants and 65 tracheal samples from NLRI participants. There were 33 (20.6%) tracheal samples missing because families missed sample collection times and because two participants died from their LRIs during the study. All 127 tracheal aspirates were analyzed via MiSeq sequencing of 16S rRNA V4 amplicons. A total of 2,743,049 sequences ranging from 1,514 to 75,719 sequences per sample (mean = 21,598.8; median = 20,727) were obtained after quality control analyses and OTU filtering. From these data, we identified a total of 950 OTUs and 10–154 OTUs (mean = 66.2) per sample (S2 Table; OTU taxa).

### The taxonomic composition of the tracheal microbiome

The tracheal microbiome across all 40 patients (127 samples) included sequences that corresponded to the following 18 genera: *Streptococcus* (16.5%), *Neisseria* (11%), *Haemophilus* (8.7%), *Moraxella* (8.1%), *Pseudomonas* (7.8%), *Corynebacterium* (6.6%), *Staphylococcus* (4.4%), *Acinetobacter* (3.3%), *Prevotella* (3%) and *Stenotrophomonas* (3%). All the other detected genera accounted for <3% of the total sequences. Each of the 127 tracheal microbiomes contained 6 to 18 (mean = 14.8 genera) of these bacterial genera. This assortment of genera reflects membership from both the oral cavity (e.g., *Prevotella*, *Streptococcus* and *Neisseria*) [27, 28, 50] and upper airways (e.g., *Streptococcus*, *Moraxella*, *Haemophilus*, *Corynebacterium*, and *Staphylococcus*) [28, 50, 51]. This is not surprising given the anatomic location of the trachea (i.e., between the upper airway and the lung).

### Tracheal microbiomes in YLRI patients differ from those in NLRI patients

**Fig 1:**
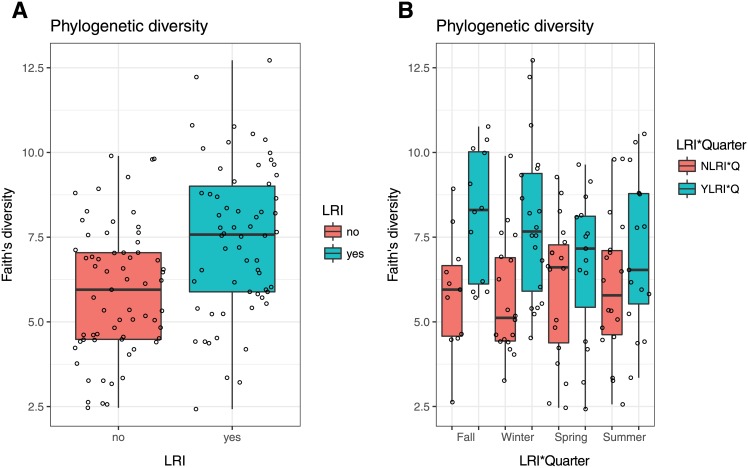
Box plots of phylogenetic alpha-diversity of microbiotas from patients with (YLRI) and without (NLRI) lower respiratory infections (LRI) (A) and of microbiotas from YLRI and NLRI patients across meteorological seasons (B).

Microbiotas from YLRI patients showed greater alpha-diversity than microbiotas from NLRI patients (Fig 1A and S1 Fig). These differences resulted significant (0.017≤P≤0.003) for three of the four indices compared in our LME analyses when accounting for steroid use (Table 2). Alpha-diversity indices also varied between YLRI and NLRI patients across quarters, particularly in Q1 (fall) and Q2 (winter) (Fig 1B); however, these differences did not result significant (P>0.05) in our LRI*Quarter LME model and analyses.

**Table 2:** Mean alpha-diversity indices and mean relative proportions of dominant phyla and genera (>3%) in decreasing order of abundance for ALL samples (NLRI+YLRI), patients with no lower respiratory infections (NLRI) and patients with lower respiratory infections (YLRI). Linear mixed-effects (LME) models results are shown for alpha-diversity indices and taxa proportions, while permutational multivariate analysis of variance (adonis) results are shown for beta-diversity indices. Significance of LME models analyses was estimated using ANOVA type II or III with Satterthwaite approximation. For each test we report the relevant F statistic (*F*), degrees of freedom (*DF*) and significance (*P(>F)*). Significant associations are indicated in bold.

Taxon	ALL	NLRI	YLRI	F	DF	P(>F)
Alpha-diversity						
ACE	75.1	64.1	87.1	6.61	39	**0.017**
Fisher	8.7	7.1	10.4	6.22	40	**0.014**
PD	6.7	5.9	7.5	9.74	40	**0.003**
Shannon	2.1	2.0	2.1	0.20	40	0.655
Beta-diversity						
Unifrac-unw				2.75	2	**0.023**
Unifrac-w				7.85	2	**0.006**
Bray-Curtis				3.40	2	**0.011**
Jaccard				2.46	2	**0.018**
Phyla						
Proteobacteria	48.8	47.1	50.5	1.28	41	0.265
Firmicutes	26.5	28.7	24.1	1.22	41	0.277
Bacteroidetes	12.5	10.6	14.5	1.11	38	0.298
Actinobacteria	8.3	9.1	7.5	2.02	42	0.163
Fusobacteria	3.2	3.5	2.9	0.65	45	0.424
Genus						
*Streptococcus*	16.5	16.7	16.2	0.69	36	0.411
*Neisseria*	11.0	9.1	13.6	2.13	37	0.153
*Haemophilus*	8.7	5.9	11.5	4.13	37	**0.009**
*Moraxella*	8.1	9.1	7.0	1.85	36	0.171
*Pseudomonas*	7.8	9.0	6.5	3.70	40	**0.025**
*Corynebacterium*	6.6	7.4	5.6	3.46	41	**0.034**
*Staphylococcus*	4.4	6.0	2.7	1.72	34	0.199
*Acinetobacter*	3.3	4.9	1.5	3.21	37	**0.030**
*Prevotella*	3.0	2.6	3.3	0.11	35	0.746
*Stenotrophomonas*	3.0	2.6	3.5	0.07	37	0.797

PCoA did not reveal clear dissimilarities in beta-diversity between YLRI and NLRI or any other variables in Table 1, since samples were not clearly depicted in discrete groups (see S2 Fig for an example). However, our adonis analyses detected significant differences (0.023≤P≤0.006) in beta-diversity between YLRI and NLRI for all of the four distances when accounting for steroid use. No significant differences (P>0.05) were observed across quarters (LRI*Quarter) in our adonis model.

**Fig 2:**
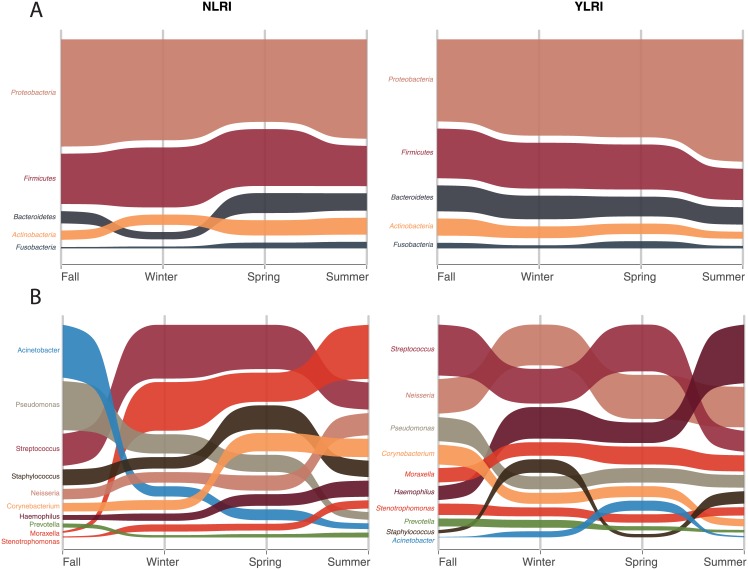
Alluvial plots of mean relative proportions of most abundant (≥3%) phyla and genera in microbiomes from patients with (YLRI) and without (NLRI) lower respiratory infections (LRI) across meteorological seasons.

Phyla abundances varied little between microbiotas from YLRI and NLRI patients (Fig 2A), but genera abundances varied more (Fig 2B). Our LME analyses showed significant associations (0.034≤P≤0.009; Table 2) with LRI for the following four bacterial genera: *Haemophilus*, *Pseudomonas*, *Corynebacterium* and *Acinetobacter*. *Haemophilus* was more abundant in YLRI patients than in NLRI patients, while the other three genera showed the inverse relationship. As before, our LME analyses did not detect significant (P>0.05) differences in taxon abundances between YLRI and NLRI samples across meteorological seasons (quarters).

## Discussion

In this study we investigated the composition and temporal dynamics of microbial communities inhabiting the trachea of tracheostomised children and young adults. Our direct tracheal sampling avoids contamination during sampling with microbes from the upper respiratory airways that might be picked up when trying to access the lower airways. We detected changes in the composition and structure of the tracheal microbiotas according to whether or not individuals developed LRI. To our knowledge this is the first study to examine the tracheal microbiome in patients with a tracheostomy. Our results provide a direct assessment of the tracheal microbiome diversity and its temporal dynamics in tracheostomised patients with and without LRIs.

Our diversity analyses show that microbiotas collected at times of health from YLRI patients had significantly higher intra-sample (alpha-diversity) diversity than the microbiotas of patients who did not develop a LRI (NLRI). All YLRI patients acquired their LRIs in the first three quarters (Q1 to Q3), while no LRI occurred during Q4 (summer). If “healthy” microbiomes were fully restored after LRI, one would expect that microbial communities in YLRI and NLRI patients would have similar levels of diversity; our results, however, seem to suggest otherwise. We suspect YLRI healthy samples are comprised of mixed-microbiotas including OTUs prevalent during “infective and healthy times”, while NLRI samples are comprised of OTUs only prevalent during health. In other words, we postulate that LRIs leave a detectable microbial signature.

The mean relative proportions of *Haemophilus*, *Pseudomonas*, *Corynebacterium*, and *Acinetobacter* varied significantly between microbiotas from YLRI and NLRI patients (Table 2). There are conflicting data about the association between *Haemophilus* and the frequency and severity of respiratory infections. Indeed, *Haemophilus* was found by Kloepfer et al. [52] to neither be associated with increased severity of rhinovirus respiratory infections, nor by Carlsson et al. [53] to be associated with the duration of wheezing in young children. By contrast, Teo et al. [43] found that infants with a *Haemophilus*-dominant nasopharyngeal microbiota had both a higher incidence and higher severity of respiratory infections. Similarly, our group found that infants hospitalized with bronchiolitis who had a *Haemophilus*-dominant nasopharyngeal microbiota at the time of hospitalization had higher rates of intensive care use and longer length of stay compared with infants who had *Moraxella*-dominant microbiota [7]. Hence, although there are discrepant findings about *Haemophilus*’ association with LRI, the weight of the current evidence suggests that the higher abundance of *Haemophilus* found in the trachea of YLRI patients is indicative of a LRI risk-microbiota [54].

The genus *Pseudomonas* is of particular importance because it includes several opportunistic human pathogens of clinical relevance. Our metataxonomic (from [55]) analyses detected 1–5 *Pseudomonas* OTUs (mean relative proportion <8%) of which OTU00005 (S2 Table) accounted for 96.7% of the 156,932 reads and was present in 90% of the patients (in 50% of them at a ≥5% mean relative proportion). BLAST searches identified this OTU as *P*. *aeruginosa* and gave it the highest E value (1e-127) in the top 1,000 references. All other *Pseudomonas* species had marginally lower E values (>1e-127). Although this result suggests that *P*. *aeruginosa* is the predominant *Pseudomonas* species, it must be interpreted with caution since 16S rRNA data cannot distinguish between different strains of *P*. *aeruginosa*. Moreover, the presence of *P*. *aeruginosa* should be validated by metagenomic approaches such as shotgun sequencing. Culture-based and microbiome analyses of bacterial communities suggest that *P*. *aeruginosa* is a common colonizer of the lower and upper respiratory airways in intubated patients [28, 56, 57]. Although *P*. *aeruginosa* may also be a devastating pathogen, especially given its frequent multidrug resistance [58]. In our study, a higher abundance of *Pseudomonas* was associated with a lower risk of developing LRI; in fact, none of the patients showed symptoms of nosocomial infections. Future work is needed to understand how *P*. *aeruginosa* transitions from a presumed asymptomatic colonizer of the lower airways to a harmful pathogen and how microbe interactions relate to disease outcomes.

*Corynebacterium* may be part of a LRI resistance-microbiota [54]. In the present study, individuals with NLRI had significantly higher abundance of *Corynebacterium* than those with YLRI. This observation is consistent with findings from Biesbroek et al. [26] who found that infants colonized with *Corynebacterium* (in addition to *Moraxella*) had a more stable microbiota over the first two years of life and fewer parent-reported respiratory infections. Teo et al. [43] also found that *Corynebacterium* dominant microbiota in the infant nasopharynx was associated with fewer respiratory infections. Furthermore, *Corynebacterium* dominance in the nasopharynx has also been associated with a reduced incidence of otitis media [59].

The genus *Acinetobacter* includes some opportunistic pathogenic species (e.g., *A*. *baumannii*) that are becoming increasingly recognized as important in nosocomial infections [60]. Moreover, *Acinetobacter* species are of increasing concern in association with ventilator associated pneumonia (VAP) [61]. In fact, a previous microbiome study by our group found representatives of this genus in the lung microbiome from mechanically ventilated patients with suspected pneumonia [56]. In our study, however, a higher abundance of *Acinetobacter* was associated with a lower risk of developing LRI; concordantly, none of the patients showed symptoms of nosocomial infections. Future work is needed to understand what and how *Acinetobacter* species become pathogenic and their interactions with other microbes during disease.

All these results combined suggest associations between community membership of specific microbial genera and health outcomes, but cause and/or effect has not been established. However, similar findings from multiple studies have helped identify a starting point for functional and/or interventional investigation that will begin unraveling the possible contributions of these bacteria to microbial network dynamics and health outcomes.

The composition (alpha-diversity) and structure (beta-diversity) of the tracheal microbiotas in tracheostomised patients with and without LRI did not change significantly over the course of one year (four meteorological seasons). Similarly, we did not find significant variation in taxon abundance between NLRI and YLRI patients across seasons. Previous studies of the nasopharyngeal microbiomes of healthy [44] and asthmatic [43] infants have revealed seasonal changes in diversity and mean relative proportions of two of the aforementioned genera (*Haemophilus* and *Corynebacterium*). A previous study by our group of the dynamics of nasopharyngeal microbiotas in asthmatic children [11] did not find differences in diversity among seasons, but found significant differences in *Haemophilus* mean relative proportions. Bogaert et al. [44] grouped fall and winter microbial samples and compared them against spring samples (no summer samples were collected), while Teo et al. [43] compared winter-fall to summer-spring samples, although no statistical justification for these groupings was provided. For the sake of comparison, we created two new variables in our study to match the seasonal groupings in those two published studies, while maintaining all the other factors in the model the same. Our LME and adonis analyses detected significant differences (0.04<P<0.01) in alpha- and beta-diversity, respectively, between NLRI and YLRI samples across seasonal groups (LRI*Seasonal Group), but not between Seasonal Groups alone (e.g., winter-fall and summer-spring). We find this outcome interesting and worth exploring, but since at this point, we do not have statistical support to group individual meteorological seasons, we advise the reader to interpret this result with caution.

Our study of the composition and temporal dynamics of microbial communities inhabiting the trachea of tracheostomised children and young adults has one main limitation. We collected samples when patients were healthy since our goal was to determine whether microbiotas from individuals who develop a LRI are different from those who do not develop a LRI. It would be also interesting to compare how the microbiomes change during heath and infection in the same individuals.

## Conclusions

We directly characterized the diversity and temporal dynamics of the tracheal microbiota in patients with and without LRIs by bypassing the nose and mouth via a tracheostomy. We demonstrated that the composition and structure of tracheal microbiotas and normalized abundances of *Haemophilus*, *Pseudomonas*, *Corynebacterium* and *Acinetobacter* differ significantly according to whether individuals developed LRIs. We also showed that tracheal microbiotas’ diversity does not change significantly over meteorological seasons. This result disagrees with previous evidence suggesting seasonal variation in airway microbiotas. Further study is needed to address the interaction between microbes and LRI during times of health and disease.

## Supporting information

S1 Table Clinical and demographic characteristics of the cohort analyzed in this study. (XLSX)

S2 Table Number of cleaned sequences per OTU and sample and corresponding taxonomic identification up to the genus level. (XLSX)

S1 Fig Box plots of Shannon, ACE, Fisher and phylogenetic alpha-diversity of microbiotas from patients with (YLRI) and without (NLRI) lower respiratory infections (LRI). (EPS)

S2 Fig 3D Principal coordinates analyses of unweighted and weighted Unifrac, Bray-Curtis and Jaccard distances between microbiotas from patients with (YLRI) and without (NLRI) lower respiratory infections (LRI). (EPS)

## References

[1] Bureau MCH. *Prevalence of CSHCN*. In: Services DoHaH, editor. Washington, DC 2008.

[2] Berry JG, Graham DA, Graham RJ, Zhou J, Putney HL, O'Brien JE, et al Predictors of clinical outcomes and hospital resource use of children after tracheotomy. Pediatrics. 2009;124(2):563–72. Epub 2009/07/15. doi: 10.1542/peds.2008-3491 ; 19596736 10.1542/peds.2008-3491 PMC3614342

[3] Dosa NP, Boeing NM, Ms N, Kanter RK. Excess risk of severe acute illness in children with chronic health conditions. Pediatrics. 2001;107(3):499–504. Epub 2001/03/07. . 11230589 10.1542/peds.107.3.499

[4] Rusakow LS, Guarin M, Wegner CB, Rice TB, Mischler EH. Suspected respiratory tract infection in the tracheostomized child: the pediatric pulmonologist's approach. Chest. 1998;113(6):1549–54. Epub 1998/06/19. . 9631792 10.1378/chest.113.6.1549

[5] McCaleb R, Warren RH, Willis D, Maples HD, Bai S, O'Brien CE. Description of Respiratory Microbiology of Children With Long-Term Tracheostomies. Respir Care. 2016;61(4):447–52. doi: 10.4187/respcare.03518 . 26670471 10.4187/respcare.03518

[6] Johnson CL, Versalovic J. The human microbiome and its potential importance to pediatrics. Pediatrics. 2012;129(5):950–60. Epub 2012/04/05. doi: 10.1542/peds.2011-2736 ; 22473366 10.1542/peds.2011-2736 PMC3340594

[7] Hasegawa K, Mansbach JM, Ajami NJ, Espinola JA, Henke DM, Petrosino JF, et al Association of nasopharyngeal microbiota profiles with bronchiolitis severity in in fants hospitalized for bronchiolitis. Eur Respir J. 2016; in press. 10.1183/13993003.00152-2016 PMC5459592 27799386

[8] Lemon KP, Klepac-Ceraj V, Schiffer HK, Brodie EL, Lynch SV, Kolter R. Comparative analyses of the bacterial microbiota of the human nostril and oropharynx. mBio. 2010;1(3). doi: 10.1128/mBio.00129-10 ; 20802827 10.1128/mBio.00129-10 PMC2925076

[9] Hilty M, Burke C, Pedro H, Cardenas P, Bush A, Bossley C, et al Disordered microbial communities in asthmatic airways. PloS one. 2010;5(1):e8578 doi: 10.1371/journal.pone.0008578 ; 20052417 10.1371/journal.pone.0008578 PMC2798952

[10] Castro-Nallar E, Shen Y, Freishtat RJ, Pérez-Losada M, Manimaran S, Liu G, et al Integrating metagenomics and host gene expression to characterize asthma-associated microbial communities. BMC Medical Genomics. 2015;8:50 26277095 10.1186/s12920-015-0121-1 PMC4537781

[11] Perez-Losada M, Alamri L, Crandall KA, Freishtat RJ. Nasopharyngeal Microbiome Diversity Changes over Time in Children with Asthma. PloS one. 2017;12(1):e0170543 doi: 10.1371/journal.pone.0170543 ; 28107528 10.1371/journal.pone.0170543 PMC5249091

[12] Pérez-Losada M, Crandall KA, Freishtat RJ. Comparison of two commercial DNA extraction kits for the analysis of nasopharyngeal bacterial communities. AIMS Microbiology. 2016;2(2):108–19.

[13] Pérez-Losada M, Crandall KA, Freishtat RJ. Two sampling methods yield distinct microbial signatures in the nasopharynges of asthmatic children. Microbiome. 2016;4:25 doi: 10.1186/s40168-016-0170-5 27306800 10.1186/s40168-016-0170-5 PMC4910261

[14] Vissers M, de Groot R, Ferwerda G. Severe viral respiratory infections: are bugs bugging? Mucosal Immunol. 2014;7(2):227–38. doi: 10.1038/mi.2013.93 . 24220300 10.1038/mi.2013.93

[15] Folsgaard NV, Schjorring S, Chawes BL, Rasmussen MA, Krogfelt KA, Brix S, et al Pathogenic bacteria colonizing the airways in asymptomatic neonates stimulates topical inflammatory mediator release. Am J Respir Crit Care Med. 2013;187(6):589–95. Epub 2013/02/02. doi: 10.1164/rccm.201207-1297OC . 23370914 10.1164/rccm.201207-1297OC

[16] Larsen JM, Steen-Jensen DB, Laursen JM, Sondergaard JN, Musavian HS, Butt TM, et al Divergent pro-inflammatory profile of human dendritic cells in response to commensal and pathogenic bacteria associated with the airway microbiota. PLoS ONE. 2012;7(2):e31976 Epub 2012/03/01. doi: 10.1371/journal.pone.0031976 ; 22363778 10.1371/journal.pone.0031976 PMC3283686

[17] Tomosada Y, Chiba E, Zelaya H, Takahashi T, Tsukida K, Kitazawa H, et al Nasally administered Lactobacillus rhamnosus strains differentially modulate respiratory antiviral immune responses and induce protection against respiratory syncytial virus infection. BMC immunology. 2013;14:40 doi: 10.1186/1471-2172-14-40 ; 23947615 10.1186/1471-2172-14-40 PMC3751766

[18] Perez-Losada M, Castro-Nallar E, Bendall ML, Freishtat RJ, Crandall KA. Dual Transcriptomic Profiling of Host and Microbiota during Health and Disease in Pediatric Asthma. PloS one. 2015;10(6):e0131819 doi: 10.1371/journal.pone.0131819 ; 26125632 10.1371/journal.pone.0131819 PMC4488395

[19] Larsen JM, Musavian HS, Butt TM, Ingvorsen C, Thysen AH, Brix S. COPD and asthma-associated Proteobacteria, but not commensal Prevotella spp.,promote TLR2-independent lung inflammation and pathology. Immunology. 2014. 10.1111/imm.12376 PMC4298427 25179236

[20] Huang YJ, Charlson ES, Collman RG, Colombini-Hatch S, Martinez FD, Senior RM. The role of the lung microbiome in health and disease. A National Heart, Lung, and Blood Institute workshop report. Am J Respir Crit Care Med. 2013;187(12):1382–7. Epub 2013/04/26. doi: 10.1164/rccm.201303-0488WS . 23614695 10.1164/rccm.201303-0488WS PMC5155250

[21] Mackenzie GA, Leach AJ, Carapetis JR, Fisher J, Morris PS. Epidemiology of nasopharyngeal carriage of respiratory bacterial pathogens in children and adults: cross-sectional surveys in a population with high rates of pneumococcal disease. BMC infectious diseases. 2010;10:304 doi: 10.1186/1471-2334-10-304 ; 20969800 10.1186/1471-2334-10-304 PMC2974682

[22] Prevaes SM, de Winter-de Groot KM, Janssens HM, de Steenhuijsen Piters WA, Tramper-Stranders GA, Wyllie AL, et al Development of the Nasopharyngeal Microbiota in Infants with Cystic Fibrosis. Am J Respir Crit Care Med. 2015 doi: 10.1164/rccm.201509-1759OC . 26492486 10.1164/rccm.201509-1759OC

[23] Cremers AJ, Zomer AL, Gritzfeld JF, Ferwerda G, van Hijum SA, Ferreira DM, et al The adult nasopharyngeal microbiome as a determinant of pneumococcal acquisition. Microbiome. 2014;2:44 doi: 10.1186/2049-2618-2-44 ; 25671106 10.1186/2049-2618-2-44 PMC4323220

[24] Allen EK, Koeppel AF, Hendley JO, Turner SD, Winther B, Sale MM. Characterization of the nasopharyngeal microbiota in health and during rhinovirus challenge. Microbiome. 2014;2:22 doi: 10.1186/2049-2618-2-22 ; 25028608 10.1186/2049-2618-2-22 PMC4098959

[25] Sakwinska O, Bastic Schmid V, Berger B, Bruttin A, Keitel K, Lepage M, et al Nasopharyngeal microbiota in healthy children and pneumonia patients. Journal of clinical microbiology. 2014;52(5):1590–4. doi: 10.1128/JCM.03280-13 ; 24599973 10.1128/JCM.03280-13 PMC3993659

[26] Biesbroek G, Tsivtsivadze E, Sanders EA, Montijn R, Veenhoven RH, Keijser BJ, et al Early respiratory microbiota composition determines bacterial succession patterns and respiratory health in children. American journal of respiratory and critical care medicine. 2014;190(11):1283–92. doi: 10.1164/rccm.201407-1240OC . 25329446 10.1164/rccm.201407-1240OC

[27] Dickson RP, Huffnagle GB. The Lung Microbiome: New Principles for Respiratory Bacteriology in Health and Disease. PLoS pathogens. 2015;11(7):e1004923 doi: 10.1371/journal.ppat.1004923 ; 26158874 10.1371/journal.ppat.1004923 PMC4497592

[28] Marsh RL, Kaestli M, Chang AB, Binks MJ, Pope CE, Hoffman LR, et al The microbiota in bronchoalveolar lavage from young children with chronic lung disease includes taxa present in both the oropharynx and nasopharynx. Microbiome. 2016;4(1):37 doi: 10.1186/s40168-016-0182-1 ; 27388563 10.1186/s40168-016-0182-1 PMC4936249

[29] Morris A, Paulson JN, Talukder H, Tipton L, Kling H, Cui L, et al Longitudinal analysis of the lung microbiota of cynomolgous macaques during long-term SHIV infection. Microbiome. 2016;4(1):38 doi: 10.1186/s40168-016-0183-0 ; 27391224 10.1186/s40168-016-0183-0 PMC4939015

[30] Graham RJ. An opportunity: critical care beyond the intensive care unit. Pediatr Crit Care Med. 2005;6(3):327–8. Epub 2005/04/29. doi: 10.1097/01.PCC.0000161286.14032.6D . 15857533 10.1097/01.PCC.0000161286.14032.6D

[31] Liptak GS, Burns CM, Davidson PW, McAnarney ER. Effects of providing comprehensive ambulatory services to children with chronic conditions. Arch Pediatr Adolesc Med. 1998;152(10):1003–8. Epub 1998/10/28. . 9790611 10.1001/archpedi.152.10.1003

[32] McPherson ML, Lairson DR, Smith EO, Brody BA, Jefferson LS. Noncompliance with medical follow-up after pediatric intensive care. Pediatrics. 2002;109(6):e94 Epub 2002/06/04. . 12042588 10.1542/peds.109.6.e94

[33] Kozich JJ, Westcott SL, Baxter NT, Highlander SK, Schloss PD. Development of a dual-index sequencing strategy and curation pipeline for analyzing amplicon sequence data on the MiSeq Illumina sequencing platform. Appl Environ Microbiol. 2013;79(17):5112–20. doi: 10.1128/AEM.01043-13 ; 23793624 10.1128/AEM.01043-13 PMC3753973

[34] Schloss PD, Westcott SL, Ryabin T, Hall JR, Hartmann M, Hollister EB, et al Introducing mothur: Open-Source, Platform-Independent, Community-Supported Software for Describing and Comparing Microbial Communities. Appl Environ Microb. 2009;75(23):7537–41. doi: 10.1128/Aem.01541-09 19801464 10.1128/AEM.01541-09 PMC2786419

[35] Schloss PD, Gevers D, Westcott SL. Reducing the effects of PCR amplification and sequencing artifacts on 16S rRNA-based studies. PloS one. 2011;6(12):e27310 doi: 10.1371/journal.pone.0027310 ; 22194782 10.1371/journal.pone.0027310 PMC3237409

[36] Edgar RC, Haas BJ, Clemente JC, Quince C, Knight R. UCHIME improves sensitivity and speed of chimera detection. Bioinformatics. 2011;27(16):2194–200. doi: 10.1093/bioinformatics/btr381 21700674 10.1093/bioinformatics/btr381 PMC3150044

[37] Wang Q, Garrity GM, Tiedje JM, Cole JR. Naive Bayesian classifier for rapid assignment of rRNA sequences into the new bacterial taxonomy. Appl Environ Microb. 2007;73(16):5261–7. doi: 10.1128/AEM.00062-07 17586664 10.1128/AEM.00062-07 PMC1950982

[38] McMurdie PJ, Holmes S. Waste Not, Want Not: Why Rarefying Microbiome Data Is Inadmissible. Plos Comput Biol. 2014;10(4). Artn E1003531 doi: 10.1371/journal.pcbi.1003531 24699258 10.1371/journal.pcbi.1003531 PMC3974642

[39] Love MI, Huber W, Anders S. Moderated estimation of fold change and dispersion for RNA-seq data with DESeq2. Genome Biol. 2014;15(12):550 doi: 10.1186/s13059-014-0550-8 ; 25516281 10.1186/s13059-014-0550-8 PMC4302049

[40] Price MN, Dehal PS, Arkin AP. FastTree 2-Approximately Maximum-Likelihood Trees for Large Alignments. Plos One. 2010;5(3). ARTN e9490 doi: 10.1371/journal.pone.0009490 20224823 10.1371/journal.pone.0009490 PMC2835736

[41] Faith DP. Conservation evaluation and phylogenetic diversity. Biol Conserv. 1992;61:1–10.

[42] Bates D, Maechler M, Bolker B, Walker S. Fitting linear mixed-effects models using lme4. Journal of Statistical Software. 2015;67(1):1–48.

[43] Teo SM, Mok D, Pham K, Kusel M, Serralha M, Troy N, et al The infant nasopharyngeal microbiome impacts severity of lower respiratory infection and risk of asthma development. Cell Host & Microbe. 2015;17:704–15. 25865368 10.1016/j.chom.2015.03.008 PMC4433433

[44] Bogaert D, Keijser B, Huse S, Rossen J, Veenhoven R, van Gils E, et al Variability and diversity of nasopharyngeal microbiota in children: a metagenomic analysis. PloS one. 2011;6(2):e17035 doi: 10.1371/journal.pone.0017035 ; 21386965 10.1371/journal.pone.0017035 PMC3046172

[45] Dixon P. VEGAN, a package of R functions for community ecology. J Veg Sci. 2003;14(6):927–30. doi: 10.1111/J.1654-1103.2003.Tb02228.X

[46] Bozdogan H. Model Selection and Akaike’s Information Criterion (AIC): The General Theory and Its Analytical Extensions. Psychometrika. 1987;52:345–70.

[47] Caporaso JG, Kuczynski J, Stombaugh J, Bittinger K, Bushman FD, Costello EK, et al QIIME allows analysis of high-throughput community sequencing data. Nat Methods. 2010;7(5):335–6. doi: 10.1038/nmeth.f.303 20383131 10.1038/nmeth.f.303 PMC3156573

[48] RDevelopmentCoreTeam. R: A language and environment for statistical computing. R Foundation for Statistical Computing, Vienna, Austria; 2008.

[49] RStudioTeam. RStudio: Integrated Development for R. RStudio, Inc, Boston, MA URL http://www.rstudio.com/. 2015.

[50] Bassis CM, Erb-Downward JR, Dickson RP, Freeman CM, Schmidt TM, Young VB, et al Analysis of the upper respiratory tract microbiotas as the source of the lung and gastric microbiotas in healthy individuals. mBio. 2015;6(2):e00037 doi: 10.1128/mBio.00037-15 ; 25736890 10.1128/mBio.00037-15 PMC4358017

[51] Charlson ES, Bittinger K, Haas AR, Fitzgerald AS, Frank I, Yadav A, et al Topographical continuity of bacterial populations in the healthy human respiratory tract. American journal of respiratory and critical care medicine. 2011;184(8):957–63. doi: 10.1164/rccm.201104-0655OC ; 21680950 10.1164/rccm.201104-0655OC PMC3208663

[52] Kloepfer KM, Lee WM, Pappas TE, Kang TJ, Vrtis RF, Evans MD, et al Detection of pathogenic bacteria during rhinovirus infection is associated with increased respiratory symptoms and asthma exacerbations. J Allergy Clin Immunol. 2014;133(5):1301–7.e3. doi: 10.1016/j.jaci.2014.02.030 . 24698319 10.1016/j.jaci.2014.02.030 PMC4047978

[53] Carlsson CJ, Vissing NH, Sevelsted A, Johnston SL, Bonnelykke K, Bisgaard H. Duration of wheezy episodes in early childhood is independent of the microbial trigger. J Allergy Clin Immunol. 2015;136(5):1208–14 e1–5. doi: 10.1016/j.jaci.2015.05.003 . 26100088 10.1016/j.jaci.2015.05.003 PMC7172099

[54] Pillai DK, Iqbal SF, Benton AS, Lerner J, Wiles A, Foerster M, et al Associations between genetic variants in vitamin D metabolism and asthma characteristics in young African Americans: a pilot study. Journal of investigative medicine: the official publication of the American Federation for Clinical Research. 2011;59(6):938–46. doi: 10.2310/JIM.0b013e318220df41 ; 21613960 10.231/JIM.0b013e318220df41 PMC3199964

[55] Marchesi JR, Ravel J. The vocabulary of microbiome research: a proposal. Microbiome. 2015;3:31 doi: 10.1186/s40168-015-0094-5 ; 26229597 10.1186/s40168-015-0094-5 PMC4520061

[56] Toma I, Siegel MO, Keiser J, Yakovleva A, Kim A, Davenport L, et al Single-Molecule Long-Read 16S Sequencing To Characterize the Lung Microbiome from Mechanically Ventilated Patients with Suspected Pneumonia. Journal of clinical microbiology. 2014;52(11):3913–21. doi: 10.1128/JCM.01678-14 . 25143582 10.1128/JCM.01678-14 PMC4313225

[57] Oliver A, Mulet X, Lopez-Causape C, Juan C. The increasing threat of Pseudomonas aeruginosa high-risk clones. Drug resistance updates: reviews and commentaries in antimicrobial and anticancer chemotherapy. 2015;21–22:41–59. doi: 10.1016/j.drup.2015.08.002 . 26304792 10.1016/j.drup.2015.08.002

[58] McCarthy K. Pseudomonas aeruginosa: evolution of antimicrobial resistance and implications for therapy. Seminars in respiratory and critical care medicine. 2015;36(1):44–55. doi: 10.1055/s-0034-1396907 . 25643270 10.1055/s-0034-1396907

[59] Laufer AS, Metlay JP, Gent JF, Fennie KP, Kong Y, Pettigrew MM. Microbial communities of the upper respiratory tract and otitis media in children. mBio. 2011;2(1):e00245–10. doi: 10.1128/mBio.00245-10 ; 21285435 10.1128/mBio.00245-10 PMC3031303

[60] Antunes LC, Visca P, Towner KJ. Acinetobacter baumannii: evolution of a global pathogen. Pathogens and disease. 2014;71(3):292–301. doi: 10.1111/2049-632X.12125 . 24376225 10.1111/2049-632X.12125

[61] Hurley JC. World-wide variation in incidence of Acinetobacter associated ventilator associated pneumonia: a meta-regression. BMC infectious diseases. 2016;16(1):577 doi: 10.1186/s12879-016-1921-4 ; 27756238 10.1186/s12879-016-1921-4 PMC5070388

